# 

**Published:** 1917-03-10

**Authors:** 


### CONTENTS.

PAGE

**The Care of the Disabled Soldier** ... ... 451

**The White City Case...** ... ... ... ... ... 452

**Hospital and Institutional News** ... ... ... 453

The Royal Infirmary, Liverpool—A Surgeon of the "Dreadnought"—The Growth of the Pay-Ward Movement—Voluntary Rationing of the Hospital Staff—Tribunals and Hospital Employees—Tuberculous Germans Interned in Switzerland—A Western Seamen's Hospital—The Plight of an Old Dispensary—The Relations of Health Visitors and Medical Men—A Curious Announcement—The Prudential Assurance Company—A Successful Year at Swansea—A Dispute about an Epidemic at Boldon Colliery—Substitutes for Glycerine—Queen Charlotte's Lying-in Hospital—Night Lights at Sanatoria—The Devon and Cornwall Homœopathic Hospital—"The Fifth" in February—Copy from Everywhere—This Week's Drug Market.

**Cerebro-Spinal Fever in the Newer Light** ... 457

The Community versus the Microbe.

**The Criminal Law Amendment Bill** ... ... 459

**Red Cross Accountancy** ... ... ... ... 460

The Policy of the Big Balance.

**Matrons' and Sisters' Appointments** ... ... 463

PAGE

**The Matrons' and Sisters' Department** ... 461

Certificated Trained Nurses and the Poor Law.

Round the Hospitals.

Nurse-Hunting Aided by Tokens—Irish Proposals to Side-Track College?—Miss Margaret Cummins and Her Staff—Homeland Working Sister Decorated—Margarine Without Grumbling—All Ranks of Women Workers Honoured.

**Some War Problems in Food** ... ... ... 464

The Patients' Meat Ration.

**The Editor's Letter-Box** ... ... ... ... 465

Mother of "a living or viable child," aged forty-eight, or over?—Hospitals and the Spirit Tax.

**The London Hospital** ... ... ... ... ... 465

Points from the Quarterly Report.

**Parliament and the Profession** ... ... ... 466

**The Nursing Problem in Ireland** ... ... ... 467

Suggested Irish Board to Grant Diplomas to Nurses.

**Royal Red Cross (Second Class)** ... ... ... 468

Awards for Valuable Services in Connection with the War.

**Passing Events and Topics** ... ... ... ... vi

**Annual Meetings** ... ... ... ... ... ... vi

**The Institutional Worker** ... ... (Suppl.) 1

The Development of the Artificial Arm.

